# Long-term evaluation of complications after osteosynthesis of the jaws in patients with head and neck trauma: an analysis from a German highest level trauma center 2007–2023

**DOI:** 10.1038/s41598-025-95455-3

**Published:** 2025-04-02

**Authors:** Ákos Bicsák, Leonie Koch, Jorit Claussen, Julia Lahmann, Lina Zeitz, Stefan Hassfeld, Lars Bonitz

**Affiliations:** 1 Clinic for Cranial- and Maxillofacial Surgery, Regional Plastic Surgery, Dortmund General Hospital, Chair of the University of Witten-Herdecke, Muensterstrasse 240, 44145 Dortmund, Germany; 2 University of Witten/Herdecke, Health Faculty, Alfred-Herrhausen-Strasse 45, 58453 Witten, Germany

**Keywords:** Facial bone fractures, Head and neck injury, Complications, Treatment protocol, Patient safety, Outcomes research, Dental trauma, Fracture repair, Risk factors

## Abstract

We present an analysis of 388 patients with major complications of a total of 13,392 hospitalized patients after osteosynthesis from our Maxillofacial Trauma Registry, which has 13,392 patients from 2007 to 2023. This retrospective study aimed to provide a detailed overview of all moderate to severe complications in head and neck injuries after surgery. The AO system and the modified Clavien–Dindo classification serve as the basis for our clinical procedure (flow chart presented). The statistical analysis included descriptive methods and χ^2^-test. In seventeen years, the complication rate was 2.9% (Clavien–Dindo class II–V). More males were involved than females (3.04:1). The most complications were found in the mandibular angle, paramedian mandible, mandibular body, and lower condylar neck areas, as well as in frontal bone, zygomatic bone, and LeFort I fractures. Adequate treatment procedures allow a low complication rate. However, areas like the mandibular angle, paramedian mandible, lower condylar neck, Le Fort I fractures, or zygomatic bone fractures remain areas with higher risk of problems, which requires further research on necessary surgical methods.

## Introduction

Surgical quality becomes increasingly important: from many more quality determining factors, the hospital length of stay, readmission, and reoperation rate depend highly on the quality of surgery and primary hospital care^1^, which we can summarize as “complications.” As we reported earlier, a low complication rate depends on adequate protocols, high-quality diagnostics, and surgery performed in a timely manner, sufficient postoperative care, and early rehabilitation^2,3^.

There are many reports on different aspects of factors related to post-surgical complications in maxillofacial traumatology: surgical approaches, use of various medications (steroids, antibiotics), and different timing or osteosynthesis methods are essential factors addressed in literature^2,4–9^. Since the initiation of modern osteosynthesis^10^, development throughout many decades has allowed us to use more accurate diagnostics, better medication protocols, and improved osteosynthesis implant systems.

Addressing those changes requires periodic review of surgical outcome on bigger patient data so that we can find trends in how these new developments affect surgical results. Our hypothesis was that using the most updated guidelines, diagnostic and therapy planning approaches, atraumatic surgery in a timely manner and up-to-date non-surgical procedures can improve patient safety and decrease the number of complications. The aim of our study was to review and classify major complications in maxillofacial traumatology in a way that a comparison to literature data is possible so that we can verify our hypothesis.

## Materials and methods

This study (No. 152/2017) has been approved by the Ethics Commission of the University of Witten—Herdecke.

This study was conducted in accordance with the Helsinki Declaration, the laws and regulations of the European Union, the Federal Republic of Germany, the State North-Rhine-Westfalia, and the General Hospital Dortmund.

### Classification of fractures

This study included patients with fractures of the head and neck region, who were treated in our department from 01.01.2007 to 30.09.2023. We used the current AO classification (AO Foundation, Davos, Switzerland)^11–15^ of facial fractures. We needed to add one more injury type: “fracture of the anterior wall of the maxillary sinus”. This entity was presented in our prior publications^16–18^.

### Clinical procedure

**Fig. 1:**
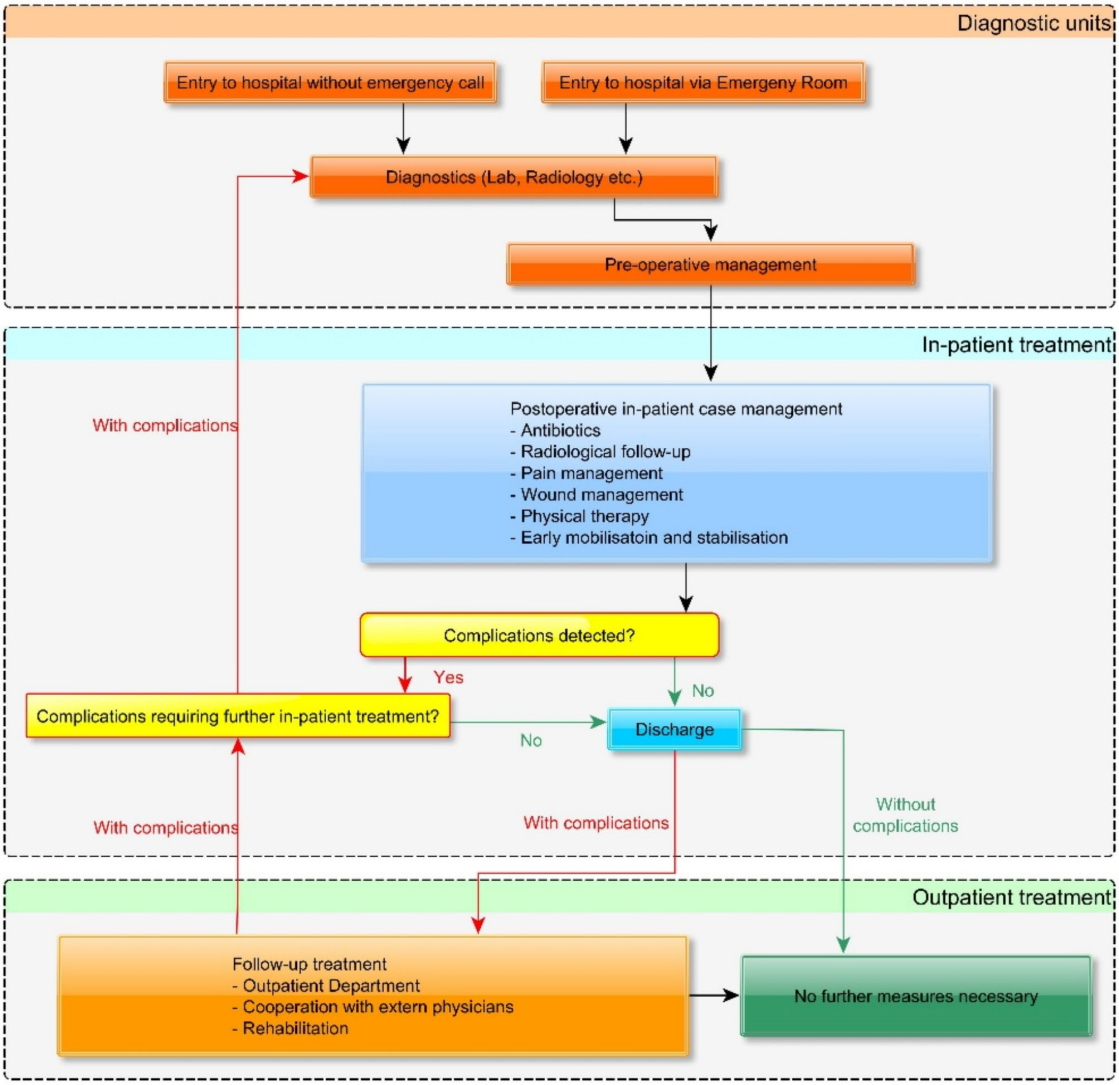
Clinical flow-chart of patient care. The patient routes are presented with arrows. Black arrows refer to all patients, green arrows to patients without complications, and red arrows to patients with complications.

Figure 1 represents the clinical procedure used for everyday patients in our Department. In the figure, we show how different organizational units can work together to minimize complications. It is essential to have an appropriate operating room unit with sufficient supplies that are available 7/24/365. Together with diagnostics, this supports inpatient care. Patients should be operated within 24 h after injury. Patients receive antibiotics (iv. ampicillin-sulbactam 3 g 3× daily or clindamycin 600 mg 3× daily), pain medication (novamin-sulfon 1000 mg up to 4× daily as per need as basis, paracetamol, oxycodone can be added as per need) and cryotherapy as standard. In case of midface fractures, oxymetazoline nasal spray or drops; in case of periorbital injuries, eye drops; in case of intraoral wounds, chlorhexidine mouth rinse are applied additionally. Patients are discharged after two days if the postoperative recovery is satisfactory. If not, the hospital stay is prolonged as needed. The antibiotic therapy ends mostly with discharge; it may be prolonged with per os medication for up to five days as per surgeons’ judgment.

Each patient undergoes a radiological follow-up. Other departments’ follow-up is organized according to need, like follow-ups with eye specialists, oto-rhino-laryngologists, etc.

### Surgery

Most surgeries are performed under general anesthesia. Standard osteosynthesis methods are used based on the AO principles using titanium osteosynthesis implants^19^ of contemporary sets with marketing authorization. The study does not consider changes made by manufacturers. If required, a mandibular fixation is applied during the surgery; the surgeon defines the time point of removal post-operatively. In most cases, the application does not exceed 2–4 weeks (as short as possible).

### Post-hospital follow-up

The follow-up after discharge was defined individually. Most patients are requested to present for at least one follow-up visit. If there are no complications, the follow-up can be performed by external specialists upon the patients’ request.

### Removal of the osteosynthesis implants

In most cases, the removal of the implants is offered to patients within four to six months after surgery, significantly if the titanium implant or the screws might disturb a potential dental rehabilitation. We discourage the removal of implants without complications in the following locations: mesh at the orbital floor and implants at the condylar neck or any further localizations potentially risking vital organs and nerve or blood vessel injury during removal^20,21^.

### Complication classification

Complications are classified as trauma-related and medical treatment-related. Each complication is defined as medical treatment-related if the complication was not present before surgery or worsened during or after surgery was observed. All complications related to injury were excluded from this analysis.

Complications are based on the modified Clavien–Dindo classification of complications in head and neck surgery^22^.

### Databank, statistics

We created the Dortmund Maxillofacial Trauma Registry. The pseudonymized data is collected in the RedCap electronic data capture system hosted by Dortmund General Hospital. The database is a web-based application enabling secure data capture, audit trail, and good interconnectivity with statistical programs^23,24^.

The statistical evaluation was performed with SPSS ver. 28.0 (IBM, US). Descriptive statistical methods to describe demographics and χ^2^-test to compare groups (level of significance: *p* < 0.05). The results are presented in the following text and figures.

## Results

**Fig. 2:**
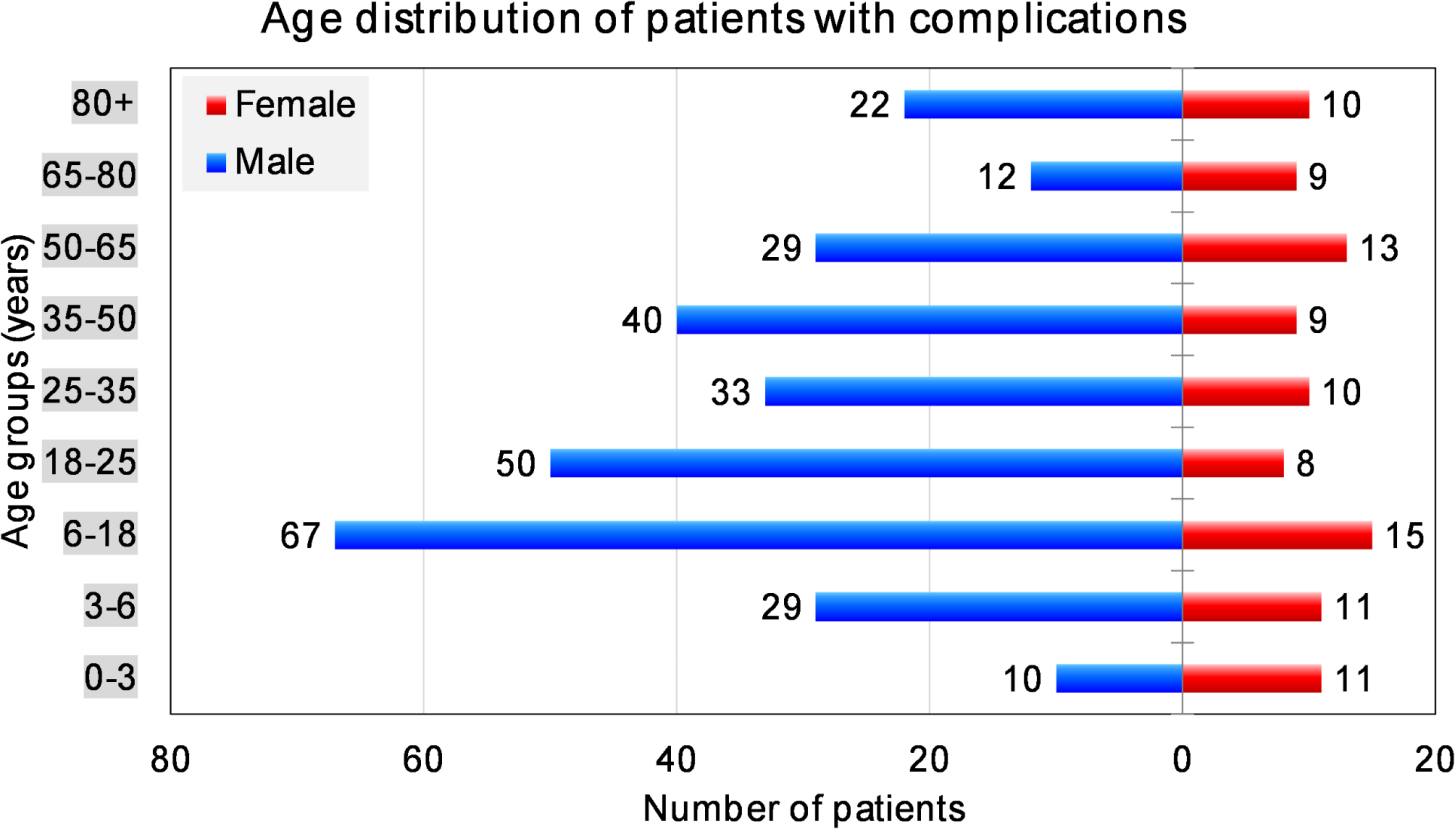
Age distribution in patients with complications.

As of 31.12.2023, the Dortmund Trauma Registry holds a total of 13,392 patients admitted to the hospital for head and neck injuries, among them 4708 females and 8684 males. Complications were observed in 96 females and 292 males making a total of 388 patients (2.9% of total). The male-to-female ratio was 1.84:1 in the total study and 3.04:1 in patients with complications. The average age in males in the total study was 38.6 y.o., in males with complications 41.7 y.o., in females respectively 52.2 y.o. and 43.8 y.o. and for the total study population respectively 43.5 y.o. and 42.2 y.o. As seen in Fig. 2, the complications distribution is nearly constant in females in all ages, while males have a clear peak in the age groups of 6–25 y.o. and a secondary peak at the age of 35–50 y.o.

Table 1 presents the summary and comparison of patient groups. The difference in the whole study between males and females is statistically significant (χ^2^-test, significance: *p* < 0.05), also in most age groups except children 0–10 y.o. (*p* = 0.757) and subjects aged 70–80 y.o. (*p* = 0.537).

**Table 1:** Presentation of the demographic results with comparison of male and female patients (χ^2^-test, significance: *p* < 0.05, significant values presented with *.

	Male	Female	Total	*p*-value
80+	22	10	32	0.006*
70–80	12	9	21	0.537
60–70	29	13	42	0.001*
50–60	40	9	49	< 0.001*
40–50	33	10	43	< 0.001*
30–40	50	8	58	< 0.001*
20–30	67	15	82	< 0.001*
10–20	29	11	40	< 0.001*
− 10	10	11	21	0.757
Total	292	96	388	< 0.001*

**Fig. 3:**
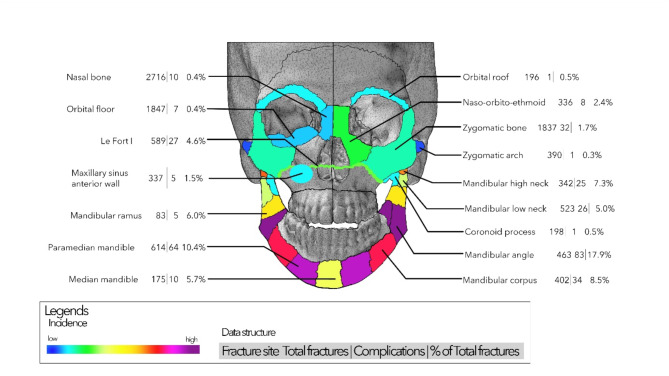
General overview of all fractures and complications in all regions of the face. The percentage value represents the percentage of the complications compared to the number of total fractures in each region. Please note that lateralization of the fractures is not provided; each number refers to fracture or complication sites. Thus, these numbers may be higher than the total number of patients.

Figure 3 presents a general overview of the detailed distribution of complications. The most complications were observed in the mandibular angle area (83 complications in a total of 463 fracture sites, 17.9%), in the paramedian mandible (64 complications in a total of 614 fracture sites, 10.4%), and in the mandibular body (34 complications in 402 fracture sites, 8.5%). In other regions, the complication rate was as high as 5% or less.

**Fig. 4:**
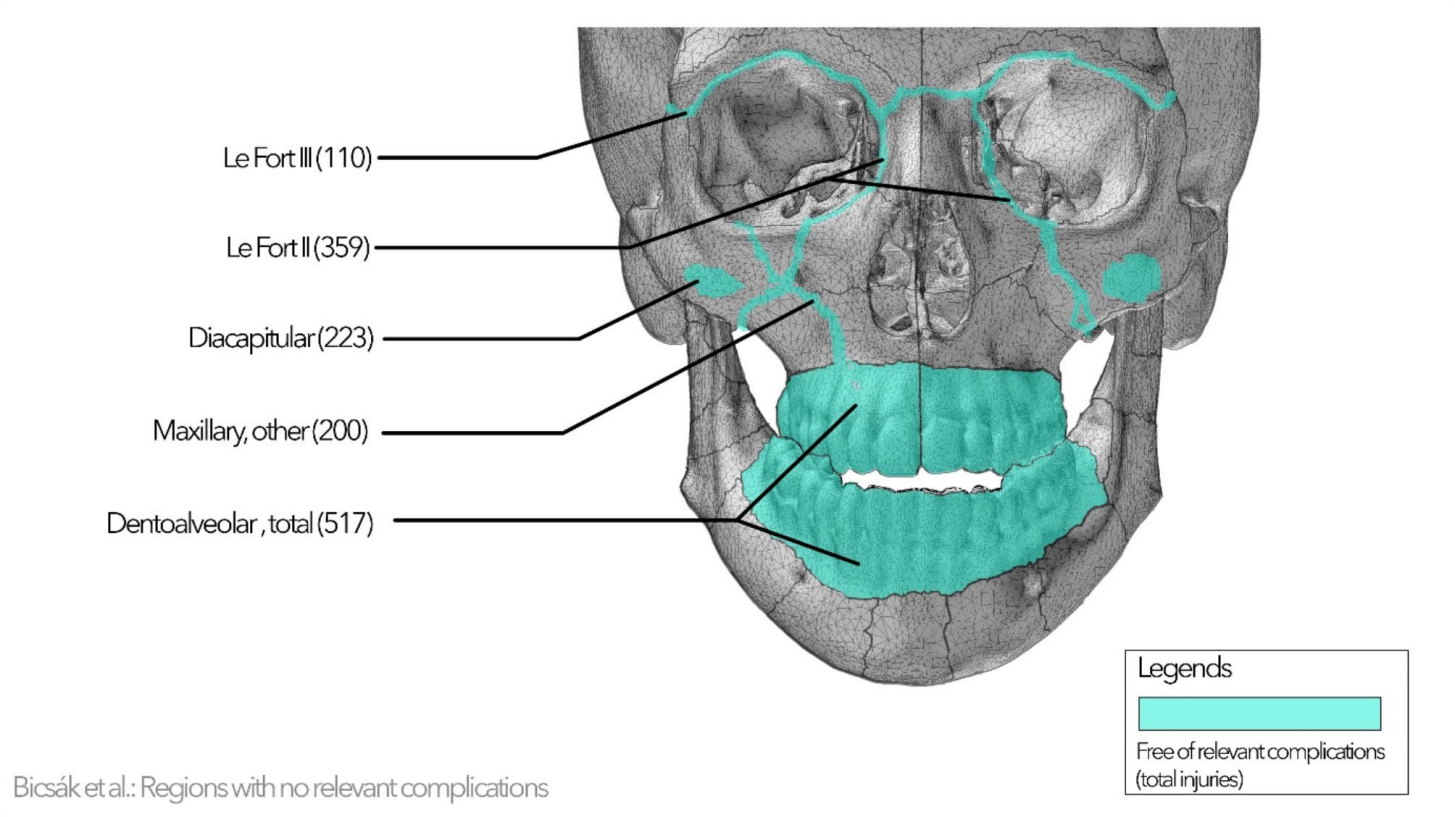
Representing the fracture site without significant complications (fracture sites that were only presented with minor fracture complications, no surgical complications that matches study inclusion criteria).

Figure 4 represents the fracture site without significant surgical complications. These are the dentoalveolar region both in the upper and lower jaw, mandibular diacapitular, Le Fort II and III fractures, and atypical maxillary fractures.

**Fig. 5:**
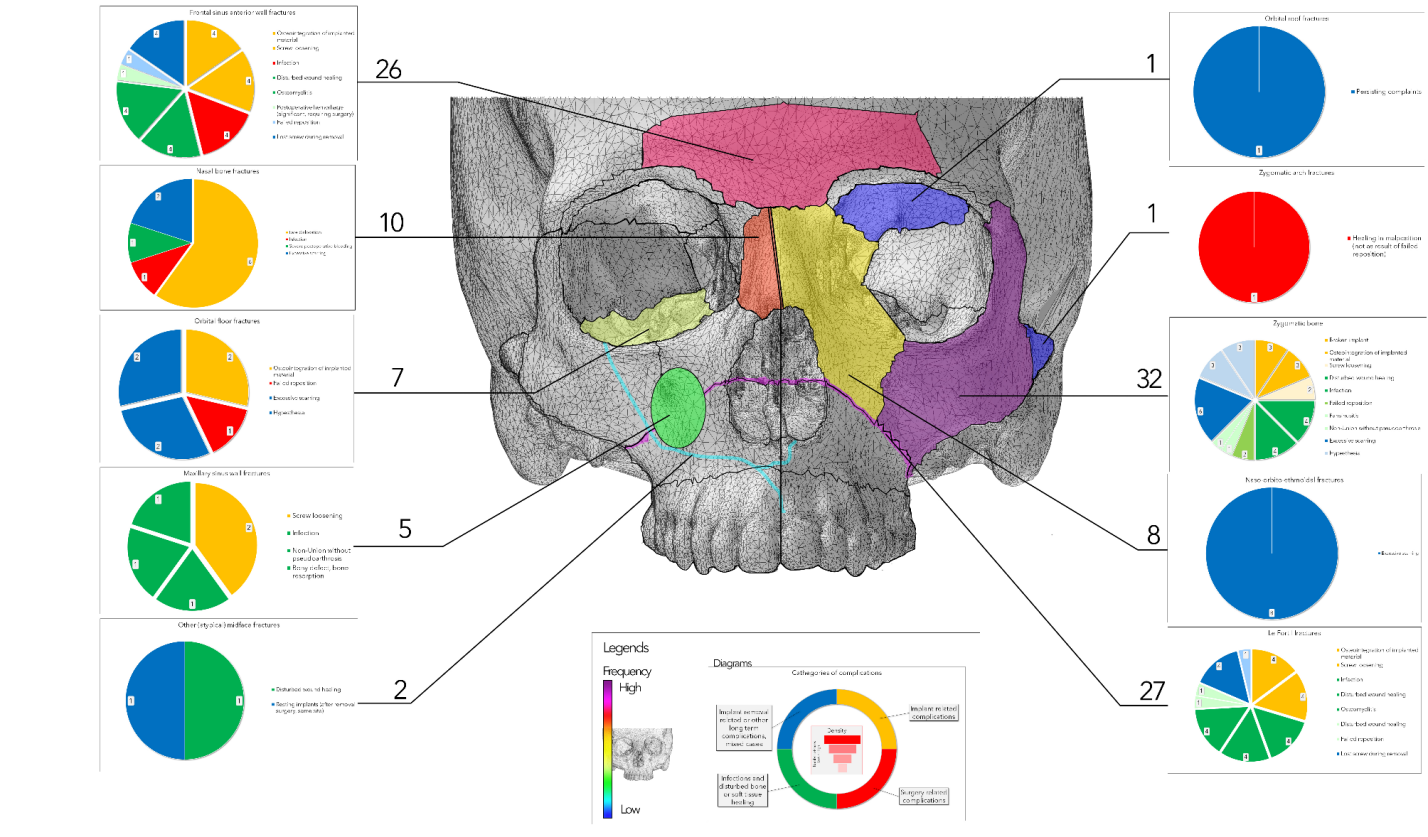
Representing the complication distribution in the frontal and midface region. Please, note that the heatmap of the skull figure refers for the marked regions only. The color scheme used in the diagrams difers also from this heatmap. To analyse the figure, please start in the center and proceed to the sides for detailed information.

In the following figures, a detailed classification of all complications is listed. The complications can be (1) implant-related, (2) surgery-related, (3) infections, soft tissue and bone healing disturbance, and (4) long-term and implant removal-related. Figure 5 shows the detailed distribution in the upper face and midface. Zygomatic bone fractures, frontal sinus wall fractures and Le Fort I fractures show the highest rate of complications. In these areas, implant-related complications and infections make up a high proportion of the complications (above 60–75%).

**Fig. 6:**
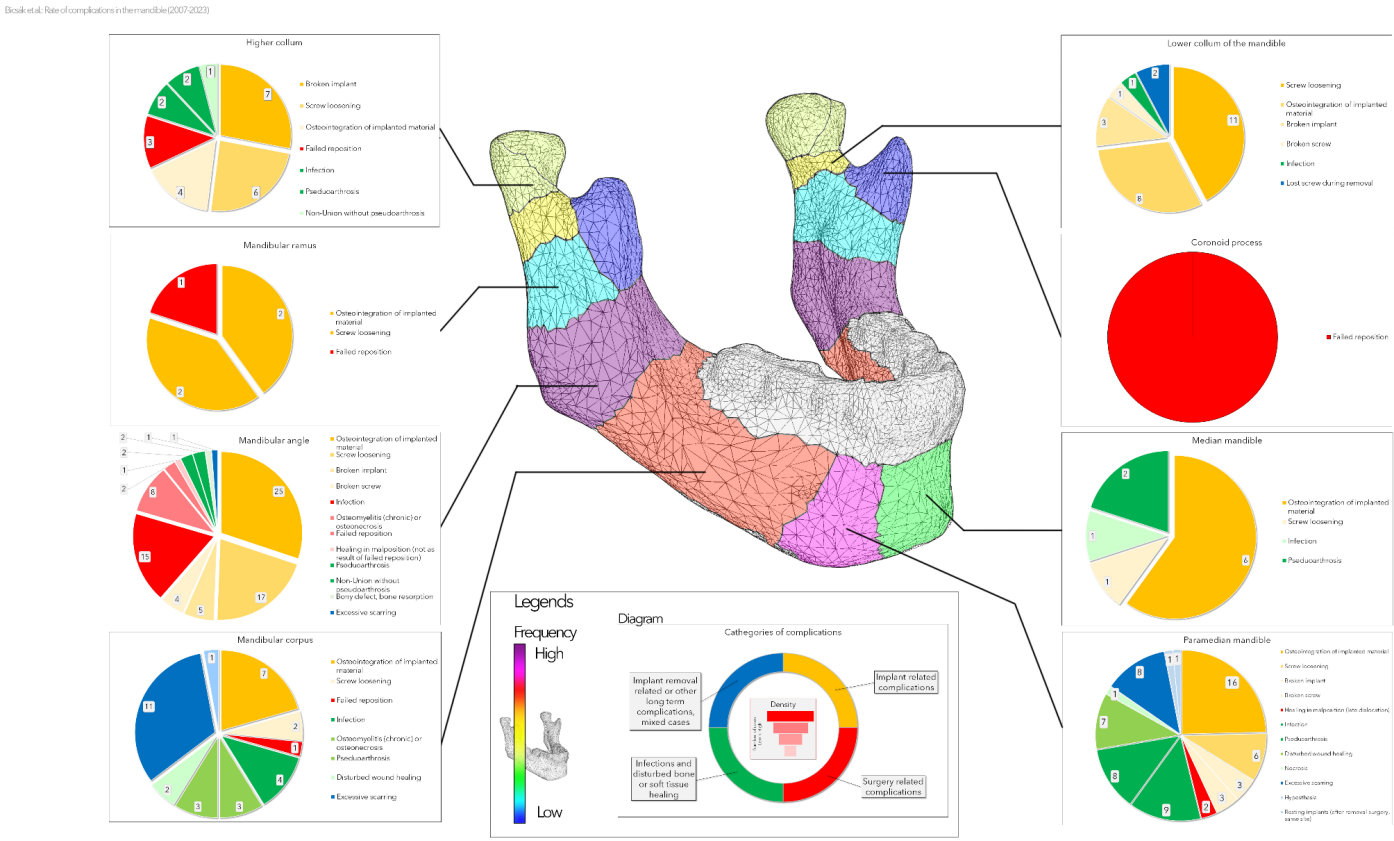
Complication distribution in the mandible. The heatmap refers to mandibular complications only. Please not the double coloring scheme on the jaw figure and on the diagrams. To analyse the figure, please start in the center and proceed to the sides for detailed information.

Figure 6 represents the same dataset for the mandibular complications. As already shown, the mandibular angle and paramedian mandible are at high risk of complications. It is a very obvious trend that infections are most seen and dominate in the paramedian and body area. In the whole mandible, the rate of implant-related and surgical complications is remarkably higher (> 50% of all complications) than in the midface area, even 88.5% (23 of 26) in the lower condylar neck. Exceptions are only the coronoid process, mandibular body and paramedian mandible.

**Fig. 7:**
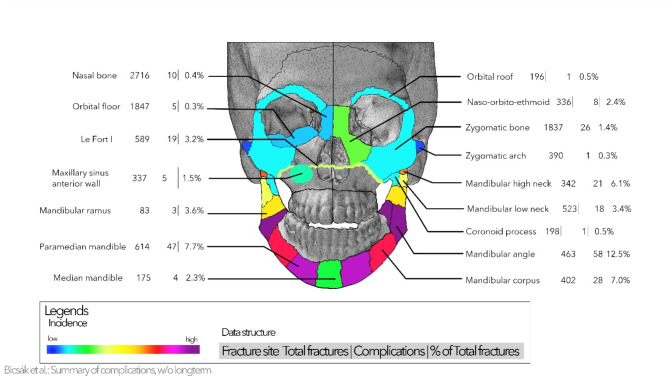
Representing the complications after exclusion of complications related to implants (marked yellow in Figs. 5 and 6 diagrams).

As many centers do not remove osteosynthesis implants, it is essential to see a clear picture without complications related to this procedure. Figure 7 represents the above corrections. Further, the mandibular angle remains the leading site (58 complications of 463 fractures, 12.5%). Also, the paramedian mandible (47 complications out of 614, 7.7%) and mandibular body (28 out of 402, 7.0%) show a high risk of complications. The complication rate decreases highly in the median mandible (4 instead of 10), lower condylar neck (18 instead of 26), and Le Fort I fractures (19 instead of 27). The comparison of Figs. 3 and 7 shows a very similar picture with minimal changes in the ranking of the regions.

## Discussion

Our study is one of the most extensive monocentric studies so far on complications after surgery for head and neck injuries. We assessed the data from 1.1.2007 to 31.12.2023 in 17 years of a total of 13,392 patients with maxillofacial injuries treated in an inpatient manner. The demography of the patients is in concordance with the literature: males are more likely to suffer injury and complications. Most authors suppose that younger males are more risk-takers than females and have the highest rates of interpersonal violence among head and neck injured^25–30^.

For the classification of complication severity, the modified Clavien–Dindo classification was used^22^. This classification matches clinical needs and is easy to apply. We decided not to analyze the surgical complications that are light and do not need special treatment. The main reason for this was that specific light fracture complications and surgical complications overlap both in the clinical picture and timely. Many of them are expected after every surgery, too. The main reason for this differentiation is that 80% of patients report sequelae after injury, 7% even persisting problems (Petersen et al.^31^).

These have no significant impact on fracture healing or long-term quality of life and do not prolong healing to a noticeable extent, as most surgeries occur within 24 h after injury. A detailed analysis and the complication rate related to specific surgical procedures are not always present. The overall complication rate in our study is 2.9%, which is, in comparison with the literature, low. Gokharman et al. found up to 48.93% for mild complications, and for severe, 4.25%^27^. Brucoli et al. stated 12.3% complications after mandibular angle fractures^32^. James et al. found complications in 42.1% of the patients after open repair of mandibular fractures^33^. Also, in this paper, 5% mechanical, 14% infections, 20% paresthesia, and 11% facial palsy were reported^33^. Juncar et al. reported 5% complications after zygomatic arch reposition, 1.6% osteitis, 2.9% malunion, and 0.5% persisting paresthesia^34^. Koirala and Subedi found 5.7% infections and 5.7% sialocele, 8.6% temporary malocclusion, 11.4% facial nerve palsy after transparotid approach for condylar fractures^35^. Kuang et al. have found up to 31.12% of wound complications in a large databank^36^. In the elderly population, Michalak et al. described 21.78% complications, among them visual and sensory disturbance, malocclusion, and infections^29^. Nayak et al. reported 6.6% overall complications in a similar treatment setup as in our study^37^, which is remarkably low, but still more than a 2-fold rate. Our results for the mandibular angle are comparable to those of Brucoli et al.^32^—12.3% and 12.5%.

Aman et al. presented a summary of secondary operations after maxillofacial trauma^4^. Unfortunately, no data on the complication rate is provided. Due to the primarily immediate surgical fixation, we see nearly no need for secondary corrections, like osteotomies. Removal of osteosynthesis implants remains an open question with all medical and financial impacts. Sukegawa et al. provided a detailed summary on implant removals^21^. This study removed osteosynthesis implants after a mean of 630 days. 4–18% of the implants were removed due to infection^21^. In comparison, we suggest removing the implants after 4–6 months (120–180 days); the overall infection rate in our patients is below this rate.

The fracture sites with the highest complications risk (mandibular angle, paramedian mandible, lower condylar neck, mandibular body, but also frontal bone, zygomatic bone, and Le Fort I plane) represent areas with complications > 5%, which we assess as high-risk areas. We think, these areas are biomechanically the most complex regions. Also, the above-mentioned mandibular areas have a higher risk of wound healing disturbance in other surgeries, not just in trauma surgery. Orbital floor traumatology seems to be better addressed with our protocol^27,38,39^. Mandibular complications appear to be similar or better to those reported by other authors^5,32,35^. In the case of mandibular fractures, the preferred usage of an intraoral approach to condylar neck and mandibular angle instead of extraoral or transbuccal approaches seems to have a good effect^35^.

The literature generally indicates that hospitalization took 4.0 days^26^ to 6.8 days^5^, but some authors indicate an increase in the case of complications. In our population, the length of stay was 3.45 days on average for primary surgery and 4.38 days for surgeries for complications. Implant removals without complications were discharged after two days.

Antibiotic use remains questionable. A recent meta-analysis by Dawoud et al. has not found strong evidence for antibiotic use in mandibular fractures. However, there were no studies without the application of antibiotics to compare. Therefore, we can say there is an implicit consensus for antibiotic administration and no reason to discard antibiotic treatment. Our protocol with three times three grams of amoxicillin-clavulanic acid IV or alternatives in case of allergy over 48–72 h until discharge seems to provide satisfactory coverage.

A meta-analysis saw steroid use as good for decreasing postoperative edema and pain^9^. Some concerns about impaired wound healing were expressed. In our experience, single steroid shots have no real negative influence. The wound infection in our study is more associated with the anatomical site, as patients receive steroids if no contraindication is present.

We have not found any similar presentation on complication details and rates at specific fracture sites. Therefore, a comparison with the literature is not possible.

The limitation of the study is its retrospective and monocentric nature. We think, a prospective design is ethically highly questionable. Monocentric studies are generally lower ranked than multicentric ones. However, this nature allows an adequate assessment of the treatment concept and other factors utilized during patient care. Randomization is also impossible within a concept, except for more minor details, like antibiotic use, etc. Therefore, we think, the above study provides the highest possible evidence without ethical or professional concerns.

Further limitations were the already-mentioned application of the Clavien–Dindo classification and the overlapping of the light fracture complications and surgical complications.

## Conclusions

Due to many different circumstances (for example area of living, patients’ will, different severity of fractures) a uniformity in follow-up treatment cannot be provided. However, if patients present to follow-up elsewhere, hey are referred to local colleagues with sufficient experience. Therefore, we can assume that patients that require high level care for their complications, are re-referred to our Department.

Our study confirms our hypothesis that surgical and non-surgical measures are essential for patient safety and successful trauma surgery of the head and neck region. In our experience and in concordance with the literature the following factors play an important role in providing a safe care in maxillofacial traumatology:

- Quick diagnostics and proper treatment planning.
- Osteosynthesis as per the AO suggestions.
- Peri- and postoperative use of antibiotics.
- Steroid use (single-shot intraoperatively).
- Early postoperative radiological follow-up.
- Close clinical follow-up.
- Early rehabilitation and discharge.

## Data Availability

The data that support the findings of this study are available from the corresponding author upon reasonable request.
